# Method for Extracting Arterial Pulse Waveforms from Interferometric Signals

**DOI:** 10.3390/s25144389

**Published:** 2025-07-14

**Authors:** Marian Janek, Ivan Martincek, Gabriela Tarjanyiova

**Affiliations:** Physics Department, Faculty of Electrical Engineering and Information Technology, University of Zilina, Univerzitná 8215/1, 010 26 Žilina, Slovakia; ivan.martincek@uniza.sk (I.M.); gabriela.tarjanyiova@uniza.sk (G.T.)

**Keywords:** arterial pulse waveform, Fabry–Perot interferometer, Python-based processing

## Abstract

This paper presents a methodology for extracting and simulating arterial pulse waveform signals from Fabry–Perot interferometric measurements, emphasizing a practical approach for noninvasive cardiovascular assessment. A key novelty of this work is the presentation of a complete Python-based processing pipeline, which is made publicly available as open-source code on GitHub (git version 2.39.5). To the authors’ knowledge, no such repository for demodulating these specific interferometric signals to obtain a raw arterial pulse waveform previously existed. The proposed system utilizes accessible Python-based preprocessing steps, including outlier removal, Butterworth high-pass filtering, and min–max normalization, designed for robust signal quality even in settings with common physiological artifacts. Key features such as the rate of change, the Hilbert transform of the rate of change (envelope), and detected extrema guide the signal reconstruction, offering a computationally efficient pathway to reveal its periodic and phase-dependent dynamics. Visual analyses highlight amplitude variations and residual noise sources, primarily attributed to sensor bandwidth limitations and interpolation methods, considerations critical for real-world deployment. Despite these practical challenges, the reconstructed arterial pulse waveform signals provide valuable insights into arterial motion, with the methodology’s performance validated on measurements from three subjects against synchronized ECG recordings. This demonstrates the viability of Fabry–Perot sensors as a potentially cost-effective and readily implementable tool for noninvasive cardiovascular diagnostics. The results underscore the importance of precise yet practical signal processing techniques and pave the way for further improvements in interferometric sensing, bio-signal analysis, and their translation into clinical practice.

## 1. Introduction

The arterial pulse is one of the fundamental diagnostic parameters in medicine, arising from the pulsatile activity of the heart [1]. The heart’s activity generates a pressure response in the arterial tree, which results from the dynamic interaction between the volume of blood ejected by the heart during systole and the ability of the vascular system to adapt to this ejected blood volume. This pressure response depends on the condition of the heart and the vascular system, and its course is characterized by the arterial pulse waveform (APW). The shape of the APW changes along the arterial tree, with an increase in pressure amplitude during systole relative to diastole on peripheral arteries compared to the aorta [2]. Analysis of the APW shape at various pulse points on the human body can provide physicians with valuable information, enabling the assessment of the overall state of the cardiovascular system in patients with cardiovascular diseases [3].

Since the analysis of the APW shape finds application in medical practice, various non-invasive sensors are being developed that can record the APW with high accuracy [4,5,6]. These sensors operate on various physical principles, which are based on measuring pressure using the piezoelectric effect [4,7], measuring elongation using the Bragg wavelength [5], utilizing the properties of ultrasound [6], the absorption and scattering of light [8], etc. Each type of sensor has its advantages and disadvantages in clinical applications. Recently, new types of optical sensors have emerged that utilize the measurement of light interference in Fabry–Perot cavities (FPC) to record the APW [9,10,11,12]. Light interference measurement can be performed in the spectral or time domain, and thus, various experimental setups for recording interference signals exist, allowing the measurement of the APW. One commonly used setup of the Fabry–Perot interferometer (FPI), which typically employs optical fibers, is called a low-finesse FPI. This type of interferometer is one of the most reliable and precise fiber-optic sensors, enabling the measurement of dynamic events with high accuracy through phase modulation of the optical signal [13,14]. Due to its advantages, such as compact design, resistance to electromagnetic interference, and high sensitivity, the low-finesse FPI is also used for rapid pressure measurement and monitoring of APWs [10,11,15]. A disadvantage of optical sensors with FPC that measure light interference in the time domain can be the fact that these sensors do not measure the APW directly. The sensors measure the time dependence of the interference signal, from which the APW needs to be determined using phase demodulation methods.

In the measurement of light interference in the time domain, the techniques of demodulating the interference signal largely determine the performance of the entire sensing system. This is also true for determining the APW by demodulating the interference signal from the low-finesse FPI, which is obtained by measurement in sensing elements using applanation tonometry. Therefore, it is crucial to develop methods for demodulating the interference signal to obtain high-quality data, enabling its use in the study of cardiovascular diseases.

This work integrates the low-finesse Fabry–Perot interferometric technique with Python-based signal processing to extract APWs. While various studies describe methods for demodulating interferometric signals, the field lacks publicly available, open-source tools to perform this task, creating a barrier to reproducibility and further innovation. A key novelty of this paper is to address this gap by providing the complete, step-by-step processing pipeline as an open-source codebase hosted on GitHub https://github.com/jamarian123/FPI-Pulse-Waveform-Extraction (accessed on 28 June 2025). By leveraging the precision of optical sensing and the versatility of Python libraries, we aim to enhance the accuracy and efficiency of signal analysis, contributing to the development of cardiovascular diagnostic tools. This work presents a multi-step procedure for extracting APWs via Fabry–Perot interferometry. Outlier removal, Butterworth filtering, min–max normalization, the rate of change, and Hilbert transform are used to identify peaks and minima even under low-amplitude. An interactive method refines breakpoint detection when the phase of the interferometric signal changes direction for accurate segmentation. The concluding results emphasize the potential of Fabry–Perot-based APW monitoring for precise cardiovascular assessment, with opportunities for enhanced sensor design and more sophisticated algorithm development.

It is known that in ideal interferometry conditions for unknown waveforms, amplitude limitations (not exceeding λ/4) and the necessity for quadrature point stabilization are important. Our approach, however, is intentionally geared toward a practical, simpler, and potentially lower-cost implementation specifically for APW extraction. Consequently, our system operates with APW displacement amplitudes that can exceed λ/4 and does not incorporate active operating point stabilization. While general fringe counting methods for large, arbitrary signals can indeed face challenges in accurately demodulating complex waveform details, our approach is specifically adapted to the known quasi-periodic nature and distinct morphological characteristics of the APW signal.

To address potential ambiguities at signal turning points, which can occur with a fixed-wavelength interferometer for such amplitudes, we employ a tailored multi-step processing strategy. This includes the robust identification of global extremes and, crucially, physiologically relevant local extrema (termed ’breakpoints’) that signify changes in the direction of membrane displacement. As described, where automated detection might be ambiguous, particularly in noisy or low-amplitude signal regions, our method incorporates an iterative refinement process. This allows for visual observation and operator-guided adjustments of these critical points, constrained by physiological understanding of the APW and boundary conditions, such as the requirement for an even number of breakpoints within a single cardiac cycle. This specialized approach enables us to successfully extract quality APW signals with detailed morphological features, as demonstrated by our results.

## 2. Materials and Methods

This section details the experimental apparatus, the principles of signal acquisition using our low-finesse Fabry–Perot interferometer, the procedures for optimizing signal quality, and the subsequent Python-based signal processing pipeline developed for extracting and analyzing APW signals.

Central to achieving reliable APW extraction is the concept of measurement specificity. In the context of our work, specificity refers to the crucial ability of our Fabry–Perot interferometric system and subsequent signal processing pipeline to selectively measure and accurately extract the target APW. Our methodology aims to enhance this specificity through the choice of a low-finesse interferometer, careful experimental tuning of sensor sensitivity, and a multi-stage Python-based processing approach encompassing outlier removal, targeted filtering, and robust feature extraction techniques, all designed to isolate the true APW dynamics from these confounding factors.

To practically realize this desired specificity and optimize the quality of the acquired interferometric signal, a key aspect of our experimental procedure involved the careful tuning of the Fabry–Perot sensor’s sensitivity. This optimization was performed experimentally with the dual objective of achieving high responsiveness to the nuanced dynamics of the APW, while simultaneously minimizing the sensor’s susceptibility to other physiological signals and environmental artifacts present within the measurement range. Efforts were specifically directed at reducing the system’s sensitivity to common interferences, such as artifacts originating from muscle activity, respiratory movements, and other subtle subject motions or mechanical vibrations, partly achieved by adequately pressing the sensor onto the relevant skin area, and by the stabilization of the body at the pulse point against a fixed surface. This approach enhanced the specificity of the APW capture and the overall quality of the recorded interferometric signal.

### 2.1. Fabry–Perot Interferometric Signal

Fabry–Perot interferometers can be broadly categorized based on their finesse, which is a measure of how sharply the device resonates. High-finesse setups typically feature highly reflective mirrors with narrow, well-defined interference fringes, making them sensitive to very small changes in optical path length but also more susceptible to alignment issues and longer response times. By contrast, low-finesse FPI systems have lower reflectivity and broader interference fringes, reducing their sensitivity to slight path length variations but providing a larger dynamic range and more forgiving alignment tolerances [13,16]. In arterial pulse monitoring, a low-finesse FPI configuration can be advantageous because it offers fewer constraints on mirror alignment, a simpler design, and robust operation over a wider range of displacement amplitudes. High-finesse setups, while more precise under ideal conditions, can be more challenging to stabilize in practical, real-world measurements of arterial pulsation. We utilize a low-finesse FPI setup, which operates on the principle of arterial applanation tonometry [11]. This method allows non-invasive measurement of the APW by applanating (flattening) a superficial artery at arterial pulse points. When using optical sensors based on FPI, the applanating of the artery is typically done using an elastic surface that forms one side of the FPC. This elastic surface of the sensor bends due to the pressure of the sensor head on the artery and the arterial pressure, causing a change in the length of the FPC. The change in the length of the FPC can be detected by measuring the interference of the optical signal reflected from the reflective surfaces of the FPC.

**Figure 1:**
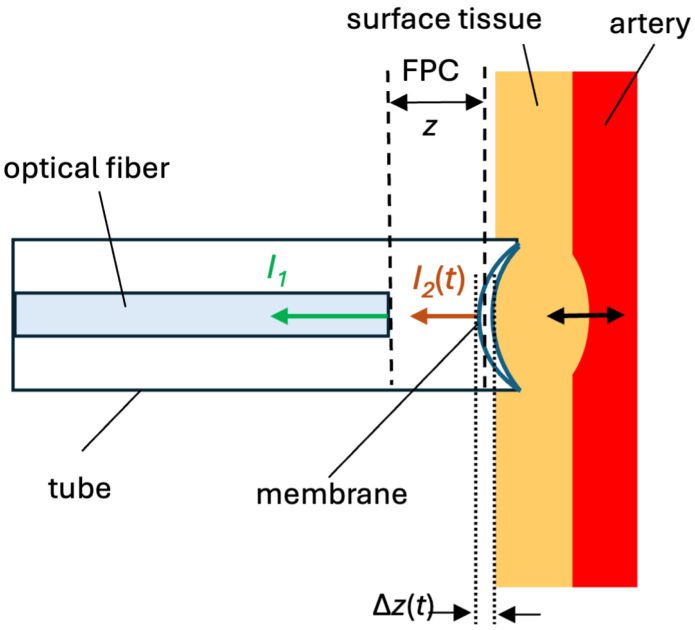
Schematic of our low-finesse Fabry–Perot cavity formed between the fiber end and a thin elastic membrane.

In Figure 1, the schematic of our low-finesse FPI is shown. The cleaved end of the optical fiber (left) and the low-reflective elastic membrane (center/right) create an FPC. Incident light from a semiconductor laser with a wavelength of 1551.3 nm is fed through an optical fiber circulator from an optical fiber with an FPC. Light in the fiber partially reflects off the fiber end, producing intensity $I_{1}$, and the light then continues to the membrane, where a second partial reflection $I_{2}\left(t\right)$ occurs. These reflections recombine, and the resulting interference pattern, which is detected by a photodiode and displayed on an oscilloscope, depends on the cavity length z±Δz(t) or just Δz(t). The membrane contacts the arterial wall on its right side, allowing the pressure changes from pulsatile blood flow to deform the membrane and thus vary the cavity length. Because this is a low-finesse FPI configuration, the reflectances at each surface are small but sufficient for measurable interference without stringent alignment, enabling robust arterial pulse measurements.

The intensity of the optical signal reflected from a low-finesse FPI, where the FPC is formed by the end of an optical fiber and a suitable low-reflective elastic membrane, can be expressed as  [11]:

(1) $$I\left(\lambda ,t\right)=I_{1}+I_{2}\left(t\right)+2\sqrt{I_{1}I_{2}\left(t\right)}\cos\left(\frac{4\pi n}{\lambda }\left(z\pm \Delta z(t)\right)+\pi \right),$$

where $I_{1}$ is the intensity of light reflected from the end of the optical fiber, $I_{2}\left(t\right)$ is the intensity of light reflected from the elastic membrane, *t* is time, *n* is the refractive index of the medium inside the FPC, λ is the wavelength of light, *z* is the length of the optical cavity, and Δz is the change in the cavity length due to the movement of the elastic membrane. When using a constant wavelength of light, the change in the intensity of the optical signal given by Equation (1) depends only on time *t*.

Assuming that the change in the intensity of light $I_{2}\left(t\right)$ reflected from the elastic membrane is small (a small change in $I_{2}\left(t\right)$ causes a small change in the amplitude of the signal I(t)), the alternating component of the intensity I(t) of the interference signal in the time domain will be proportional to:

(2) $$I\left(t\right)∼\cos\left(\frac{4\pi n}{\lambda }\left(z\pm \Delta z(t)\right)+\pi \right)=\cos\left(\Delta \Phi \left(t\right)+\Phi_{0}\right),$$

where

(3) $$\Delta \Phi \left(t\right)=\pm \frac{4\pi n}{\lambda }\Delta z\left(t\right)$$

represents the temporal change in the phase of the signal I(t). From Equation (2), it is clear that the intensity I(t) has a cosine waveform dependent on the temporal change in phase ΔΦ(t), which changes due to the movement of the elastic membrane. The signal I(t) is periodic in certain regions with a phase change period of 2π. When the phase of the signal I(t) changes by 2π, one interference fringe is added to I(t).

**Figure 2:**
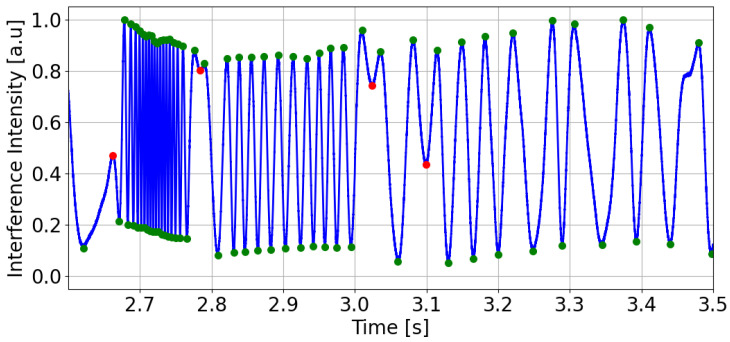
A typical interference signal I(t) for our low-finesse FPI. Green symbols indicate extrema, and red symbols denote extrema associated with transitions in the cardiac cycle.

A typical interference signal I(t) for a low-finesse FPI measured by the arterial applanation tonometry method at an arterial pulse point at a single wavelength is shown in Figure 2. The green symbols represent the extremes, and their overall density relates to the steepness or the rate of change in the reconstructed arterial pulse. The red symbols appear where the pulse wave changes direction, effectively subdividing each heartbeat into its key phases: the start of systole (first red symbol), the end of systole (second red symbol), the dicrotic notch (third red symbol), the start of diastole (fourth red symbol), and the end of diastole, which coincides with the onset of the next systolic phase.

The signal in the figure has an oscillatory waveform, with the frequency of oscillations changing in different parts of the signal. The signal starts with high-frequency oscillations, which correspond to the increase in arterial pressure during cardiac systole. In applanation tonometry with optical FPI sensors, this results in a decrease in the length of the FPC due to the bending of the elastic membrane of the FPC, which is reflected in the measured signal as alternating global maxima and minima, with the phase of the signal changing by 2π between two maxima (or minima). The number of these maxima and minima depends on the positioning of the sensor head at the arterial pulse point, and the greater their number, the higher the accuracy of determining the APW obtained by demodulating the signal I(t). After reaching the maximum systolic pressure, whose peak is characterized by a local extremum in the interference signal, the frequency of oscillations decreases as the arterial pressure gradually decreases. Regarding the bending of the elastic membrane of the FPI, when the maximum systolic pressure is reached, its bending stops, and the membrane begins to move in such a way that the length of the FPC increases. The change in the bending of the membrane, where the decrease in the length of the FPC changes to an increase, is reflected in the signal I(t) by the aforementioned local extremes. Several such local extrema, where the increase in the length of the FPC changes to a decrease or vice versa, can be identified in the I(t) signal, and they usually determine significant points on the APW, such as the start of systole, peak systole, dicrotic notch, and end of diastole.

The method of demodulating the signal I(t) measured by arterial applanation tonometry using an optical FPI and determining the APW is described in the following subsections of the paper.

### 2.2. Normalization of Measured Signal

**Figure 3:**
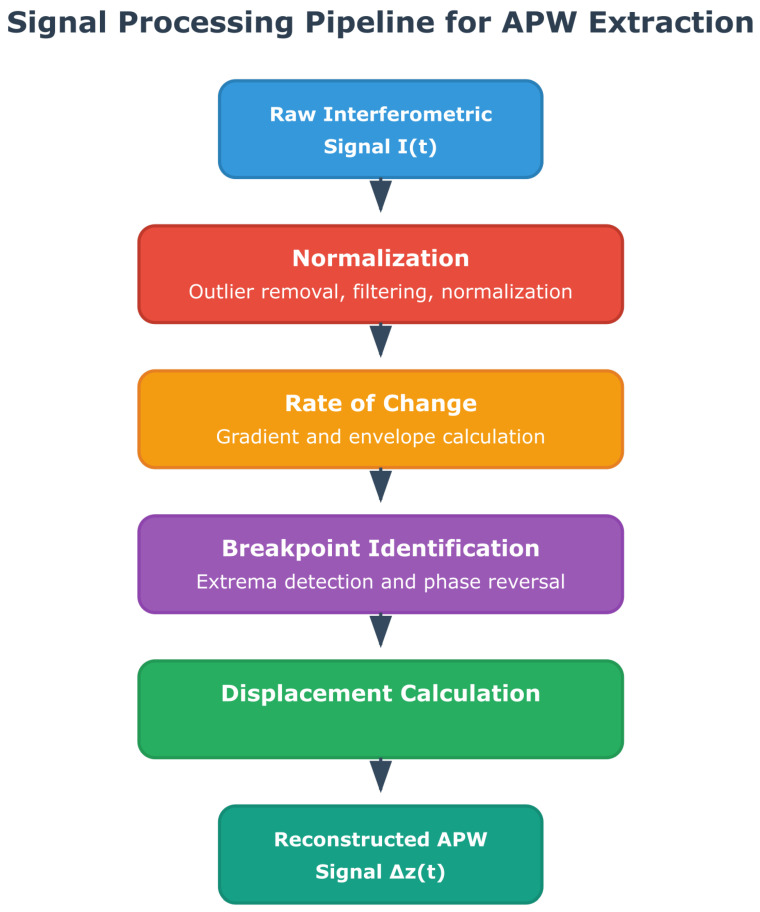
Schematic diagram briefly summarizing the entire data processing.

The entire data processing, which is detailed in the following subsections, can be summarized by the schematic diagram shown in Figure 3. As a central contribution of this work, the scripts corresponding to each of these steps are provided in our open-source repository to allow for full reproducibility and community use.

The script https://github.com/jamarian123/FPI-Pulse-Waveform-Extraction/blob/main/001-base-file-check-with-normalization.py (accessed on 28 June 2025) begins by verifying that the input file contains the required structure, numeric data, and consistent time steps. Next, it identifies outliers (strength can be adjusted) in the signal via Z-scores, which quantify how far values deviate from the mean in terms of standard deviations [17,18]. Any detected outliers are subsequently replaced using interpolation, combined with forward and backward filling, to preserve the continuity of the signal. A fourth-order Butterworth filter with a 0.5 Hz cutoff is then applied to remove low-frequency noise while retaining essential higher-frequency components [19]. Then, the script segments the data into 1-second intervals and performs min–max normalization, mapping each segment’s values to a [0, 1] range for consistent amplitude representation.

Jitter (small, rapid, and undesired variations in the signal) arises from noise in optical components (e.g., light source instability), mechanical vibrations in the FPI setup, and electrical noise in photodetectors or data acquisition systems, as well as physiological factors such as natural variability in arterial pulsation (e.g., heart rate variability) and motion artifacts from the subject or sensor placement. Jitter is very well handled at the lower frequencies, where it is effectively suppressed due to low-pass filtering. On the other hand, at high frequencies, small variations do not significantly affect the signal extremes.

Filtered and normalized signals are suitable for subsequent analyses, and raw and preprocessed signals are saved in the CSV file (preprocessed.csv). By integrating validation, filtering, and normalization, the script produces a robust, high-quality dataset that supports reliable interpretation.

### 2.3. Finding the Rate of Change of Signal

The script https://github.com/jamarian123/FPI-Pulse-Waveform-Extraction/blob/main/002-roc_epoch.py (accessed on 28 June 2025) analyzes preprocessed time-series signals by focusing on the rate of change to extract a smoothed envelope to identify peaks. The rate of change of a signal provides an immediate measure of how rapidly the signal’s amplitude is changing at each time point. Practically, the rate of change can be computed as the first derivative or discrete gradient of the waveform, highlighting sharp transitions or slopes in the signal. After computing the rate of change, the Hilbert-transform is applied to derive its analytic signal, from which the instantaneous amplitude or “envelope” can be extracted [20,21]. This envelope emphasizes the underlying modulation profile of the rate of change, making it easier to detect features such as peaks, valleys, or low-amplitude segments, even in the presence of noise. By combining the rate of change and the Hilbert transformed envelope, we can gain a robust method for highlighting subtle oscillatory behavior and demarcating critical points in the waveform.

**Figure 4:**
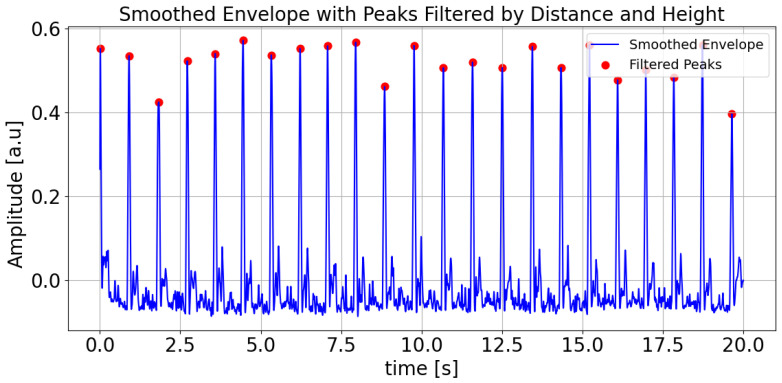
Smoothed envelope with filtered peaks by distance and height. The figure shows the smoothed envelope of the signal (blue line) with detected peaks (red dots) that meet specific distance and height criteria.

**Figure 5:**
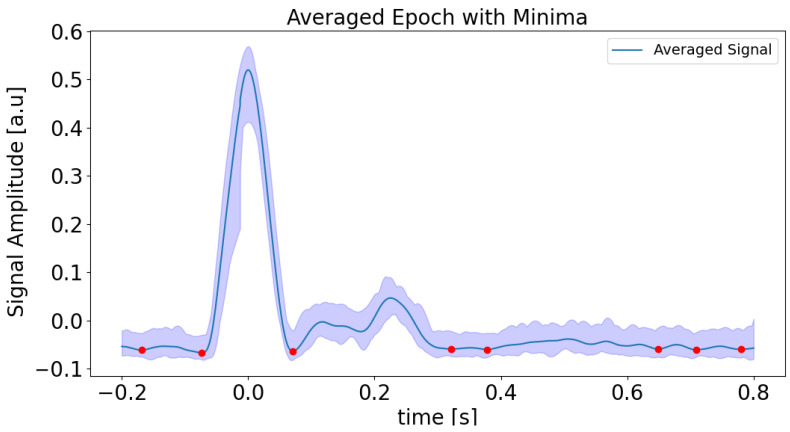
Averaged signal with detected minima and confidence interval. The figure illustrates the averaged signal (solid blue line) with a 95% confidence interval (shaded blue area) and highlights the detected minima (red points). Several such local extrema can be identified in the signal. These can be related to possible breakpoints.

Detailed steps are as follows. Processing begins by loading preprocessed data and discarding initial and final samples to mitigate edge effects. The rate of change is calculated from the signal’s gradient, after which a Hilbert transform is used to derive the normalized envelope. A Butterworth bandpass filter is then applied to smooth this envelope, which is particularly effective in low-amplitude regions. Peaks in the smoothed envelope are detected using height and distance criteria (see Figure 4), ensuring robust identification of significant features while reducing noise. Each detected peak serves as an event onset for epoch analysis, then the signal is segmented and extracted within a defined time window around the peak using the neurokit2 software 0.2.11 package [22]. Averaging these segments produces a mean signal and its 95% confidence interval, from which minima are determined via inverted peak detection (see Figure 5). These minima are then mapped to absolute times of the original signal, allowing localization of minima across the entire time range. All results, including absolute minima times and amplitudes, are visualized and saved to a CSV file for further analysis. The purpose of this step is to obtain minima or low rates of change regions, which are connected with a possible change in the phase of the interferometric signal, which is related to the change from rising to falling or vice versa in the APW signal. In our case, the first labeled minimum (on the left side of the plot in Figure 5), and the last three minima were removed because no local minima were detected in the interferometric data. This reflects the fact that the rate of change amplitude is low in these segments, corresponding to the relaxation phase in the diastolic region.

### 2.4. Breakpoint Identification

In the FPI setup [11], a thin membrane responds dynamically to arterial pulsations by expanding or contracting in rhythm with the heartbeat. Whenever the displacement of this membrane shifts from increasing to decreasing (or vice versa), the phase of the reflected signal changes its direction of progression. Such transition points in the interference signal are internally called breakpoints.

The Python 3.13.1 script marks the data points at which the membrane deflection reverses, causing a sign change in the displacement gradient. From a physiological standpoint, these breakpoints often align with key cardiac events (systolic upstroke of the APW, dicrotic notch, and diastole) that influence arterial pressure, making them important for accurately segmenting the APW into discrete cycles. A detailed description follows.

**Figure 6:**
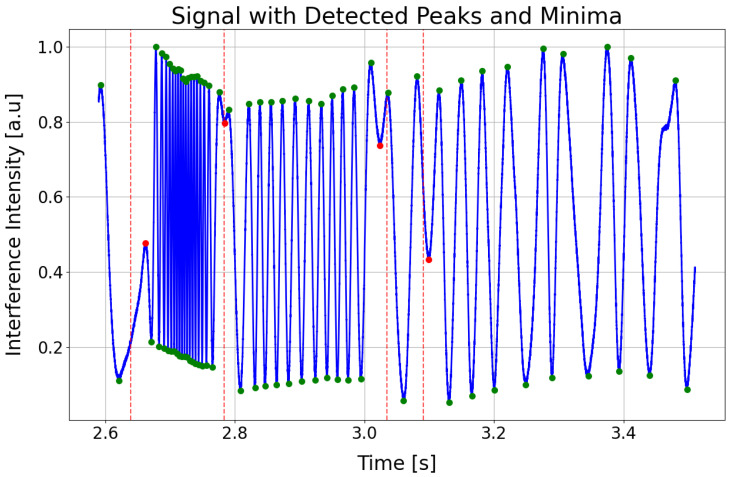
A segment of the time evolution of the interference signal, highlighting critical points for analysis. Red and green symbols denote interference extrema. Dashed red lines represent the statistically estimated positions of breakpoints, and the found breakpoints are marked by red symbols.

The script https://github.com/jamarian123/FPI-Pulse-Waveform-Extraction/blob/main/extr-breakp-sel.py (accessed on 28 June 2025) processes time-series interference data to identify and analyze local extrema (peaks and minima) and breakpoints within a specified time range. The beginning and ending parts of the signal can be cut. It begins by reading the input signal data from a CSV file, which incorporates precomputed minima times stored in a separate file (see previous subsection) for refined detection. Vertical dashed red lines, representing the low-amplitude regions of the envelope (see Figure 6), are added to highlight points of interest. The script offers interactive controls for manually adjusting any automatically detected extrema and breakpoints, using mouse clicks and keyboard shortcuts. A dynamic slider enables zooming and panning through the designated time window, making inspection and fine-tuning of the detection results more straightforward. Once confirmed, the indices of all detected extrema and breakpoints are combined, categorized as MAX (peak), MIN (minima), or BP (breakpoint), and stored in a structured CSV file. Finally, the script ensures that each analysis period begins with an extrema followed by a breakpoint, thus preserving the correct starting point (start of systole).

The purpose of this step is to obtain time positions of extremes and possible breakpoints (to find times at which the APW signal changes from rising to fall or vice versa) to reconstruct the APW signal.

### 2.5. Displacement Calculation

It begins by reading a CSV file containing the normalized interferometric data, which has a “Status” column to mark breakpoints (BP), maxima (MAX), and minima (MIN). For each row, the script calculates Δz.

Between successive amplitude extremes, the cavity length changes by approximately 386.7nm. This is because the phase difference between successive extremes is π, and the relationship between phase difference and cavity length is given by (see Equation (3)):

(4) $$\Delta z_{1}\left(t\right)=\frac{\lambda }{4n},$$

where wavelength λ=1551.3nm and refractive index of air n=1.003. If we introduce an integer variable *m*, the halfwave number, which starts at 1 after the first breakpoint and signifies the beginning of a heart-pulse period (systole), then the halfwave number increases by 1 until a breakpoint is reached, then decreases by 1 until next the breakpoint is reached, when again it starts increasing. Thus, Δz(t) can be written as,

(5) $$\Delta z\left(t\right)=m\cdot \frac{\lambda }{4n}.$$

**Figure 7:**
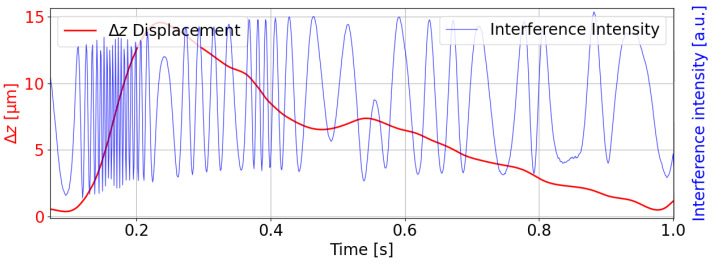
Time evolution of the displacement for one pulse (red curve), with the blue curve representing the measured interference intensity.

Using cubic spline interpolation, the script recalculates Δz at breakpoints based on nearby minima or maxima, thus creating a smooth displacement curve. The resulting columns for “m,” “delta_z,” and “I2_t” are then saved to a new CSV file and visualized using Matplotlib 3.10.0, as shown in Figure 7. The measurements were taken at the radial artery pulse point.

The figure illustrates the interpolated displacement of the FPC over a short time window (2.6–3.4 s), derived from the detected extremes (peaks and minima) and breakpoints of the interferometric signal. The displacement range reflects the high sensitivity of the FPI to arterial pulsations. Such minute displacements align with the system’s ability to detect sub-nanometer changes in cavity length. The displacement over the time window suggests a focus on the systolic upstroke of the APW, with clear oscillatory behavior indicating the dicrotic notch and subsequent diastole.

**Figure 8:**
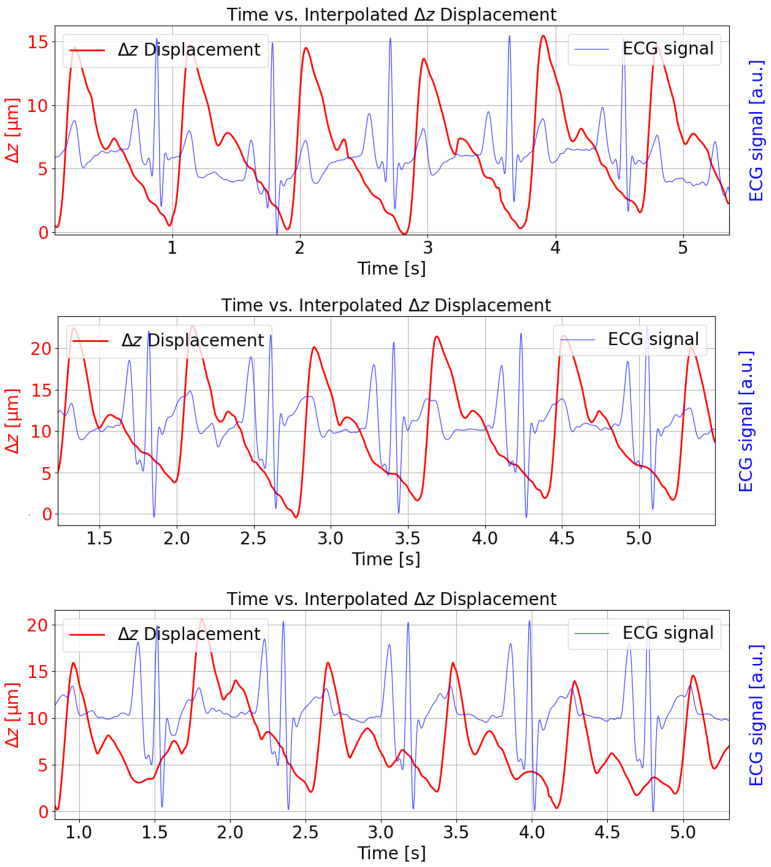
Reconstructed FPC length change (Δz) with simultaneous measurement of ECG signal over time (approx. 5 s) for three subjects. The periodic waveform reflects arterial pulsations. The measurements were taken at the radial pulse point.

Figure 8 illustrates the reconstructed FPC length change (Δz) in micrometers over approximately 5 s intervals for three different subjects (red curve). The measurements were taken at the radial artery pulse point and show the simultaneously recorded and synchronized ECG signal for temporal comparison (blue curve). The temporal alignment is consistent with physiological expectations; the ECG R-peak consistently precedes the upstroke of the arterial pulse waveform, reflecting the pulse transit time [23]. The solid red trace shows a repeating, pulse-like waveform obtained by applying cubic spline interpolation through the demodulated displacement data. Each cycle corresponds roughly to one heartbeat, with minima and maxima reflecting the elastic membrane’s motion under arterial pressure. Amplitude peaks for the displacement are in the range of 15–20 μm, and the waveform is visibly periodic, illustrating how the FPI signal recovers the arterial pulse profile over time. The repeating waveform exhibits a frequency of roughly 1 Hz, which is indicative of heartbeat oscillations.

The oscillating pattern can also be related to blood pressure variations. When the heart pumps, it momentarily increases the pressure in the arteries, causing a slight expansion (and subsequent contraction) of arterial walls. These changes are the mechanical displacements of the membrane that our system probably detects. As blood pressure rises and falls with each heartbeat, the resulting waveform in the time-series data naturally reflects this pulse pressure cycle. So, these small fluctuations in peak-to-peak intervals or amplitudes can then be attributed to the typical moment-to-moment variability in cardiovascular function, commonly observed in both heart rate and blood pressure readings.

## 3. Results and Discussion

The application of the integrated low-finesse Fabry–Perot interferometric sensing of our novel, open-source Python-based signal processing pipeline, as detailed in Section 2, consistently yielded APWs of high clarity. A quantitative measure of this performance is the signal-to-noise ratio, which was determined to be in the range of 26 to 30 dB for the extracted APWs. Such a favorable signal-to-noise range is crucial for the robust detection of detailed morphological features within the pulse wave such as the systolic upstroke, dicrotic notch, and diastolic characteristics, thereby supporting the precision required for subsequent analysis and enhancing the reliability of noninvasive cardiovascular assessment derived from the extracted waveforms. The SNR was computed using the standard formula: $SNR\left(dB\right)=20\log_{10}\left(A_{signal}/A_{noise}\right)$, where $A_{signal}$ is the root mean square (RMS) amplitude of the arterial pulse waveform, and $A_{noise}$ is the RMS amplitude of the noise.

To formally quantify the accuracy of our algorithm, we performed an error analysis using synthetic data. First, we generated synthetic ground truth APW signals with known morphological features. These waveforms were then used to simulate the expected interferometric signal, to which we added varying levels of white noise. Our complete processing pipeline was then applied to these synthetic interferometric signals. By comparing the timing of key features in the reconstructed APW against the known ground truth, we quantified the temporal accuracy of our method. The analysis demonstrated a mean temporal error in detecting extrema of less than 3 ms, confirming the robustness of our interpolation and breakpoint identification process.

The system’s high sensitivity to sub-nanometer displacements, while advantageous for resolving arterial pulsations, also renders it susceptible to motion artifacts. Even minor movements (e.g., breathing, limb adjustments) can introduce mechanical vibrations, distorting the interference signal. During applanation tonometry, the inconsistent contact pressure between the sensor and artery due to motion alters the cavity length (Δz), leading to erroneous phase (ΔΦ) calculations (not seen in Figure 7). Motion artifacts in the 0.5–5 Hz range (typical for physiological tremors) may overlap with the APW’s spectral components, complicating noise suppression via Butterworth filtering. Artifacts manifest as false peaks/minima in the rate of change envelope (practically not seen in Figure 4) or misaligned breakpoints, degrading APW segmentation accuracy. The script https://github.com/jamarian123/FPI-Pulse-Waveform-Extraction/blob/main/extr-breakp-sel.py (accessed on 28 June 2025) allows manual correction of detected features, but this requires manual user intervention.

The low-finesse Fabry–Perot design relaxes mirror alignment tolerances compared to high-finesse systems, but critical alignment challenges persist. The applanation tonometry method assumes perpendicular sensor placement relative to the artery. Angular deviations alter pressure distribution, affecting membrane deformation and Δz(t) fidelity. Poor alignment reduces signal-to-noise ratio, exacerbating interpolation errors in displacement calculations.

All potential artifacts related to alignment, angular deviations, and thermal effects were addressed. Due to the brief duration of measurements (approximately 2 min), sensor alignment and angular positioning relative to the artery were continuously visually monitored in real time. Thermal expansion effects are negligible due to the short measurement window and stable ambient temperature. Future directions can be associated with developing FPI sensors with more ergonomic housings to minimize slippage.

Sensor misalignment is observed in some regions of the data, which is dealt with by normalization; however, the signal-to-noise ratio remains unaffected. A few regions exhibit jitter, and there are questionable breakpoints located at extremes, making them difficult to distinguish from true extremes. These data points are processed manually. To determine whether a phase change occurs, applying a condition based on the pairwise number of breakpoints within a single cardiac cycle is helping.

## 4. Conclusions

APWs serve as critical indicators of cardiovascular health, encapsulating vital information about vascular compliance, cardiac efficiency, and systemic blood pressure dynamics. This work demonstrates the successful integration of the low-finesse Fabry–Perot interferometry with an advanced Python-based signal processing technique to extract and analyze APWs.

A significant contribution of this work is making this entire processing pipeline publicly available as an open-source repository on GitHub, addressing a notable gap in the availability of practical tools for researchers in this field. The developed methodology, featuring a multi-step Python-based preprocessing pipeline (outlier removal, Butterworth filtering, min–max normalization), ensures high-quality signal acquisition. The subsequent rate of change analysis and Hilbert transform application facilitate accurate feature extraction, enabling reliable detection of minima (potential changes of phase direction) even in low-amplitude conditions. Interactive breakpoint identification precisely segments APW cycles by tracking phase reversals in FPI signals, aligning with physiological events such as systole, the dicrotic notch, and diastole. The system achieves a cavity length resolution of 386.7nm (distance between two successive extremes) from these phase-derived displacement calculations. These technical achievements, realized through accessible Python-based tools and computationally efficient signal processing, demonstrate the practical viability of this low-finesse FPI approach. This viability is evidenced by successful APW extraction from measurements on three subjects, where the reconstructed waveforms showed correct physiological alignment with simultaneously recorded ECG signals. Consequently, the system offers a promising pathway towards precise, non-invasive, and potentially cost-effective cardiovascular diagnostics, capable of capturing detailed APW morphology, including the systolic upstroke, dicrotic notch, and diastolic decay.

Future work on FPI can be directed to solve limitations related to the FPI system, as well as refining the computational method. The filtration of high-frequency noise from high-frequency signals and differentiating jitter from true extremes are crucial for future code development to achieve more precise APW extraction.

To advance the current methodology, future efforts will be concentrated on achieving full automation of the signal processing pipeline, thereby removing the need for manual correction of breakpoints. We will investigate advanced computational strategies to meet this goal, including the development of robust adaptive algorithms designed to minimize manual oversight. Furthermore, we plan to explore the application of machine learning techniques for the automated extraction of key features and the reliable detection of breakpoints directly from the interferometric signal. The successful implementation of these strategies is expected to significantly enhance both the scalability of our approach for analyzing large datasets and the overall reproducibility of the results.

## Data Availability

Data are contained within the article.
